# Textile waste reinforced cementitious composites: state of the art and future perspectives

**DOI:** 10.1007/s11356-026-38099-8

**Published:** 2026-08-01

**Authors:** Alessandra Tonin Incerti, Giovani Jordi Bruschi, Eduardo Pavan Korf

**Affiliations:** Graduate Program in Environmental Science and Technology, UFFS: Universidade Federal da Fronteira Sul, Rio Grande Do Sul, Erechim, 99700-970 Brazil

**Keywords:** Textile waste, Cementitious composites, Cement-based materials, Mechanical properties, Sustainable construction, Circular economy, Recycling

## Abstract

The growing textile waste from fast fashion and high consumption has increased the search for ways to reduce landfill disposal and promote circular material flows. This review analyzes the state of the art of cementitious composites reinforced with textile waste, encompassing different matrices, fiber formats, and processing strategies, as well as evaluating the mechanical, physical, and environmental parameters reported in the literature. A search in the Scopus and Web of Science databases from 2015 to February 2026 resulted in 23 articles after screening. The studies indicate an expressive increase in publications from 2021 onward, with Spain and Portugal emerging as the main contributors. Fiber incorporation generally reduces compressive strength (CS) due to increased porosity and the relatively weak fiber–matrix interface. Conversely, consistent improvements are observed in flexural strength (FS), toughness, post-cracking capacity, and thermal performance, particularly in laminated composites. Strategies such as the use of supplementary materials, fiber pre-treatments, and microstructural optimization effectively mitigate strength loss and improve bonding and durability. Environmental assessments highlight potential CO_2_ reductions and contributions to the circular economy. Despite these advances, important gaps remain, including the need for long-term durability studies, full-scale evaluations, and comprehensive life cycle assessments. Overall, findings indicate a promising field, although further advances are still required to expand its applications in the construction sector.

## Introduction

Traditional waste management practices in the textile and apparel industry remain strongly dependent on landfilling and incineration (Huang et al. 2024; Tang 2023). In contrast, the adoption of integrated recycling systems can avoid up to 10 tons of CO_2_-eq per ton of textile waste treated when compared with conventional disposal routes (Zamani et al. 2014). In addition, textile fibers account for approximately 35% of primary microplastics found in the oceans (Boucher and Friot 2017; Niinimäki et al. 2020), and the industry is responsible for approximately 20% of global industrial water pollution, mainly due to chemical dyeing and finishing processes (Ellen MacArthur Foundation 2017; Sharma et al. 2025; Niinimäki et al. 2020). This scenario is further aggravated by the high compositional variability of textile waste, which hinders its separation and recycling (Gorreta et al. 2025).

The circular economy has been associated with the Sustainable Development Goals (SDGs), and textile recycling has been identified as a relevant strategy for achieving targets related to responsible consumption, waste management, and innovation (Karmakar et al. 2025). Therefore, improving recycling routes and adopting solutions capable of extending the service life of textile waste, reintroducing it into new production cycles and reducing waste generation throughout the value chain, is essential (Huang et al. 2024). The search for sustainable and innovative alternatives has led researchers to investigate different pathways for the valorization and reuse of these materials (Baloyi et al. 2023; Tripathi et al. 2024). Technologies aimed at the recovery, recycling, and reuse of textile fibers are essential not only to mitigate their environmental impacts but also to recover their economic value and provide more efficient alternatives to disposal (Rahman et al. 2022).

The construction sector, in turn, is one of the sectors exerting the greatest environmental pressure, owing to its extensive consumption of resources such as materials, energy, and water. The adoption of more sustainable materials and the incorporation of recycled waste has therefore become key strategies for reducing its impacts (Norouzi et al. 2021; Briga-Sá et al. 2013). Globally, an estimated 36% of all solid waste originates from this sector (Alhawamdeh et al. 2024). Concrete, the second most consumed material worldwide, has an estimated annual production of 25 to 30 billion tons. This production scale generates considerable impacts: the cement production chain accounts for approximately 8–9% of anthropogenic CO_2_ emissions and consumes approximately 2–3% of global energy (Monteiro et al. 2017; Klee 2009).

In view of the gradual depletion of natural aggregates, the increasing demand for conventional materials, and the rising costs of extraction and transportation, the search for more sustainable construction solutions has intensified (Behera et al. 2014; Briga-Sá et al. 2013). Recent studies have explored the use of waste materials as inputs for the production of concrete and mortars, contributing to reduced extraction of natural aggregates and mitigation of environmental liabilities (Sambucci and Valente 2021).

Although advances in this field are promising, there is still a lack of standardization in research findings, particularly regarding incorporation methods, the nature of the textile waste used, and the resulting effects on the properties of cementitious composites. In addition, previous reviews have addressed the topic from different perspectives. Rahman et al. (2022) presented a comprehensive study on sustainable applications of textile waste in construction and geotechnical engineering, considering technical, economic, and environmental aspects. Tran et al. (2021) reviewed the use of textile waste as fiber reinforcement in cementitious composites, focusing on recycling routes, engineering properties, and reinforcement mechanisms. Sasi et al. (2024) focused on the incorporation of recycled polymeric fibers into concrete, with emphasis on structural performance. More recently, Shahriar et al. (2025) expanded this discussion by integrating mechanical performance, durability, and microstructural aspects of concrete reinforced with recycled textile fibers.

Although previous reviews have made significant contributions to the advancement of this topic, none has addressed, in an integrated manner, the bibliometric evolution of the field, the influence of different textile waste incorporation formats on cementitious composites, and the environmental implications of this valorization route. Therefore, the present study proposes a more comprehensive and up-to-date analysis, bringing together these different aspects within a unified perspective.

## Method

This study presents a literature review on the use of textile waste from the textile and apparel industry, including pre-consumer waste, post-consumer waste, and household textiles, incorporated into cementitious matrices as part of the composite.

The bibliographic survey was based on scientific articles retrieved from the Scopus and Web of Science databases, which were selected due to their broad coverage of peer-reviewed scientific publications and rigorous indexing standards, contributing to the reliability and quality of the data used in the analysis. The search included publications indexed between 2015 and February 2026. The selected period covers recent studies on the incorporation of textile waste into cementitious materials and enables an up-to-date analysis of the development of this topic. In addition, selection criteria related to document type, namely scientific articles, and language, namely English, were adopted. The search terms used are presented in Table 1.

**Table 1:** Search strategy used in the Web of Science and Scopus databases

Variable keywords	Boolean operator	Fixed keywords	Search within	Scopus	Web of Science
“Textile waste”	AND	“Cementitious composite” OR “cement-based material” OR “concrete”	Article title, keywords, and abstract	35	32
“Recycled textile”	AND	“Cementitious composite” OR “cement-based material” OR “concrete”	Article title, keywords, and abstract	12	11
“Fabric waste”	AND	“Cementitious composite” OR “cement-based material” OR “concrete”	Article title, keywords, and abstract	3	4
“Textile waste fiber”	AND	“Cementitious composite” OR “cement-based material” OR “concrete”	Article title, keywords, and abstract	7	4

**Fig. 1:**
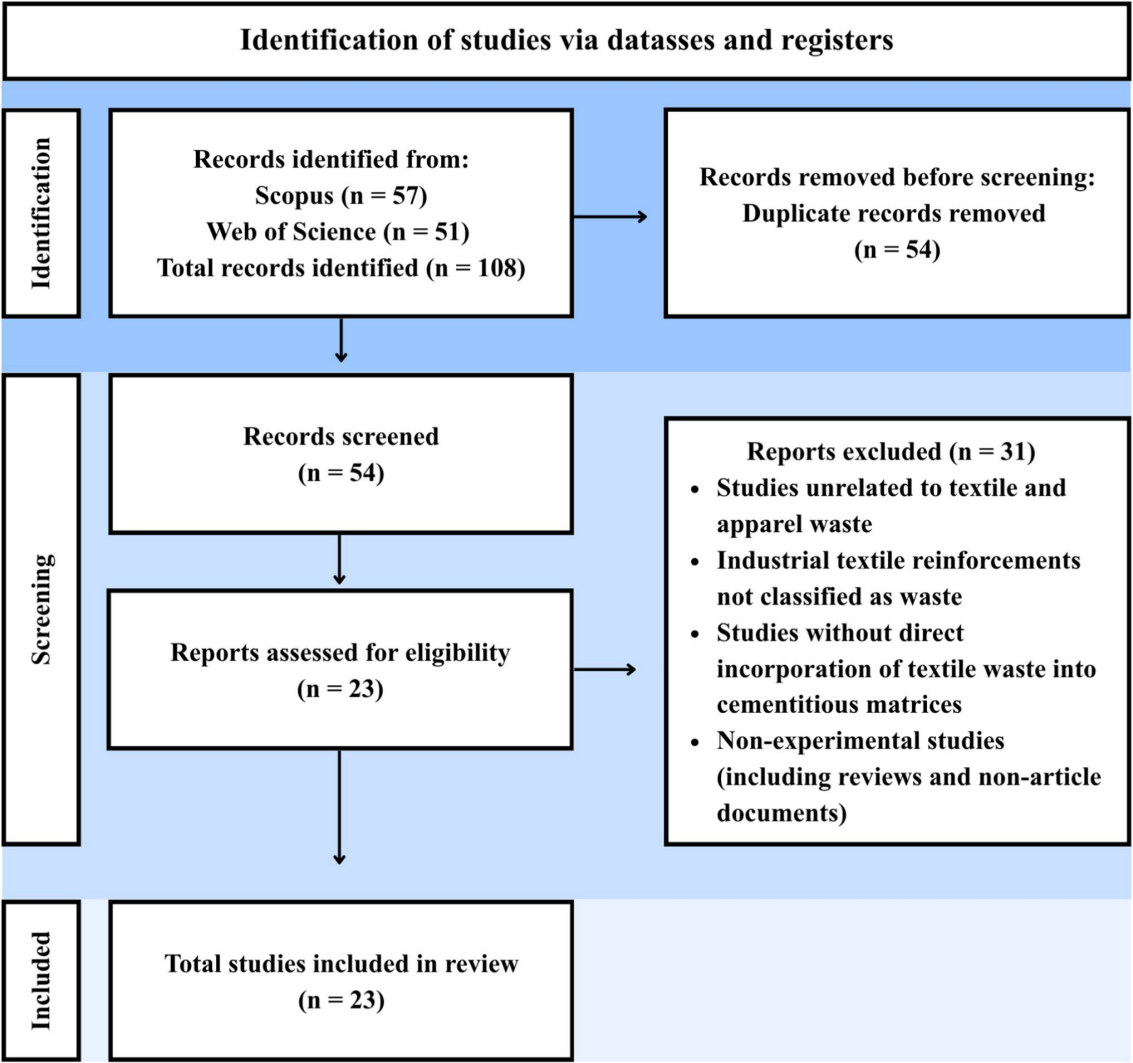
Study selection flow diagram based on the PRISMA guidelines

After merging the databases, 108 records were identified. The entire data processing procedure was performed in RStudio environment (version 4.5.2), including the automatic removal of duplicate records, resulting in a dataset of 54 studies for the screening stage. In addition, a selection flow diagram based on the Preferred Reporting Items for Systematic Reviews and Meta-Analyses (PRISMA) guidelines (Page et al. 2021) was adopted to document and visualize the process of study identification, eligibility assessment, and inclusion, as shown in Fig. 1.

The studies were screened by reading titles and abstracts and, when necessary, by conducting a full-text assessment. The eligibility criteria considered the alignment of each study with the scope of the review, methodological clarity, and the completeness of the reported experimental results.

The categorization of the studies considered parameters extracted from the selected articles, including the type of cementitious composite used, the type and origin of the textile waste, the waste format, pre-treatment procedures, method of incorporation into the cementitious matrix, curing time, and mechanical properties evaluated, particularly compressive strength (CS) and flexural strength (FS). The organization of these data enabled the development of comparative tables and a systematic synthesis of the main findings reported in the literature. Environmental assessments and future research perspectives presented in the studies were also analyzed.

In addition to the review of the selected studies, the methodology also included a bibliometric analysis based on the records identified through the database search after duplicate removal. The records were imported into RStudio software (version 4.5.2), and the bibliometric analyses were performed using the Bibliometrix package (version 5.1.1) and its Biblioshiny interface. The bibliometric results were compiled and visually presented through graphs and maps in the “Literature review” Section, accompanied by their respective interpretations.

## Literature review

Initially, 108 records were identified in the Scopus and Web of Science databases. After duplicate removal, 54 studies were retained for the bibliometric analysis. The results presented in this section provide an overview of publication trends and the geographical distribution of scientific production related to the incorporation of textile waste into cementitious composites.

### Annual scientific production

**Fig. 2:**
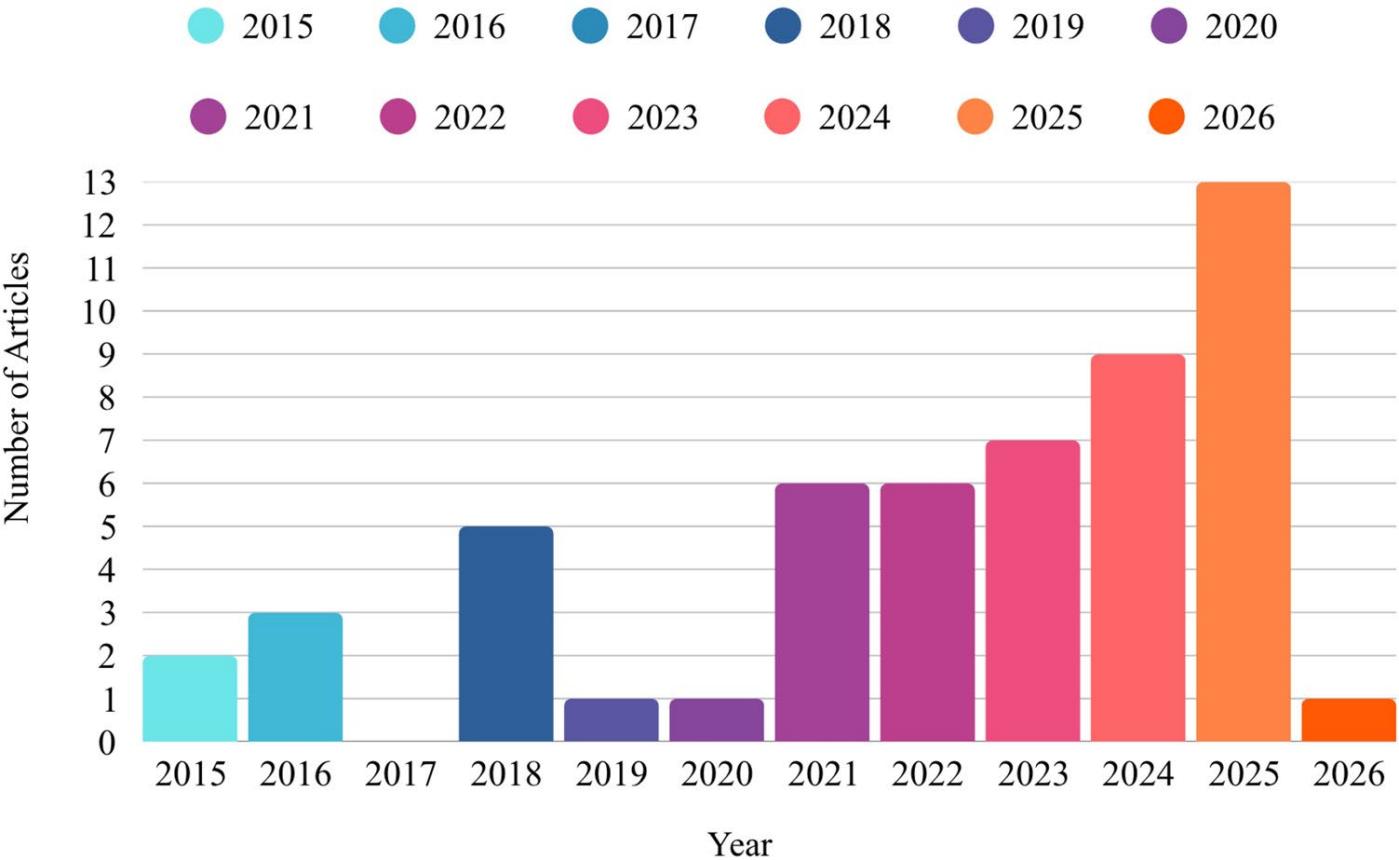
Annual scientific production of articles on textile waste incorporated into cementitious composites, based on the initial selection after duplicate removal. Note: For 2026, only articles published up to February were considered

To visualize recent publications, Fig. 2 presents the annual scientific production on the use of textile waste in cementitious composites. The search covered the period from 2015 to February 2026. In addition to the temporal distribution of publications, the studies were analyzed according to the main research trends. The data show variations in publication volume over the years, allowing the evolution and progressive consolidation of this topic in the scientific literature to be assessed.

Between 2015 and 2017, two publications were identified in 2015, three in 2016, and no records were found in 2017. The studies retrieved during this period showed limited alignment with the scope of the present review, mainly addressing technical textiles, polymer composites, and industrial waste from other production chains. This scenario indicates that, in the initial years, the application of textile waste in cementitious composites was still a poorly explored research area.

In 2018, the number of publications increased to five, followed by a decrease in 2019 and 2020, with one article published in each year. From 2018 onward, a greater convergence of publications with the scope of this review can be observed, with the emergence of the first studies focused on the incorporation of textile waste into cementitious composites. Between 2019 and 2020, the field showed gradual development, marked by the expansion of applications in cementitious composites and methodological advances in the studies.

From 2021 onward, greater regularity was observed in the number of published studies, with six articles in 2021 and six in 2022. In 2023, seven publications were identified, while 2024 and 2025 presented nine and thirteen articles, respectively, representing the years with the highest number of publications identified. By February 2026, one article had already been identified in the analyzed databases.

In 2021, a more consistent increase in scientific production was observed, accompanied by greater alignment of the publications with the scope of this review. During this period, fibrous waste from the textile and apparel industry applied in cementitious composites for non-structural building elements became particularly prominent. In addition, the studies began to broaden their focus to durability, ductility, toughness, and post-cracking behavior, indicating greater scientific maturity in the field.

Between 2022 and 2024, a significant expansion of publications was observed, with diversification in the textile wastes investigated, the applications considered, and the properties evaluated. In addition to mechanical performance, studies began to include functional analyses, such as thermal and acoustic properties, fire resistance, and durability. At the same time, sustainability-related aspects gained greater relevance, initially associated with energy efficiency and subsequently expanded to quantitative assessments of environmental impacts and carbon emissions.

In 2025 and 2026, the field reached a stage of increasing maturity, with studies focused on the development of higher-performance cementitious composites and more advanced applications. During this period, environmental and economic aspects gained prominence, with increasing application of Life Cycle Assessment and carbon emission analyses, highlighting a transition toward research oriented to technical, environmental, and economic optimization.

Overall, the results indicate a marked increase in scientific production in recent years, especially from 2021 onward, reflecting the growing interest of the scientific community in incorporating textile waste into cementitious materials and the progressive consolidation of this research field.

### Scientific production by country

**Fig. 3:**
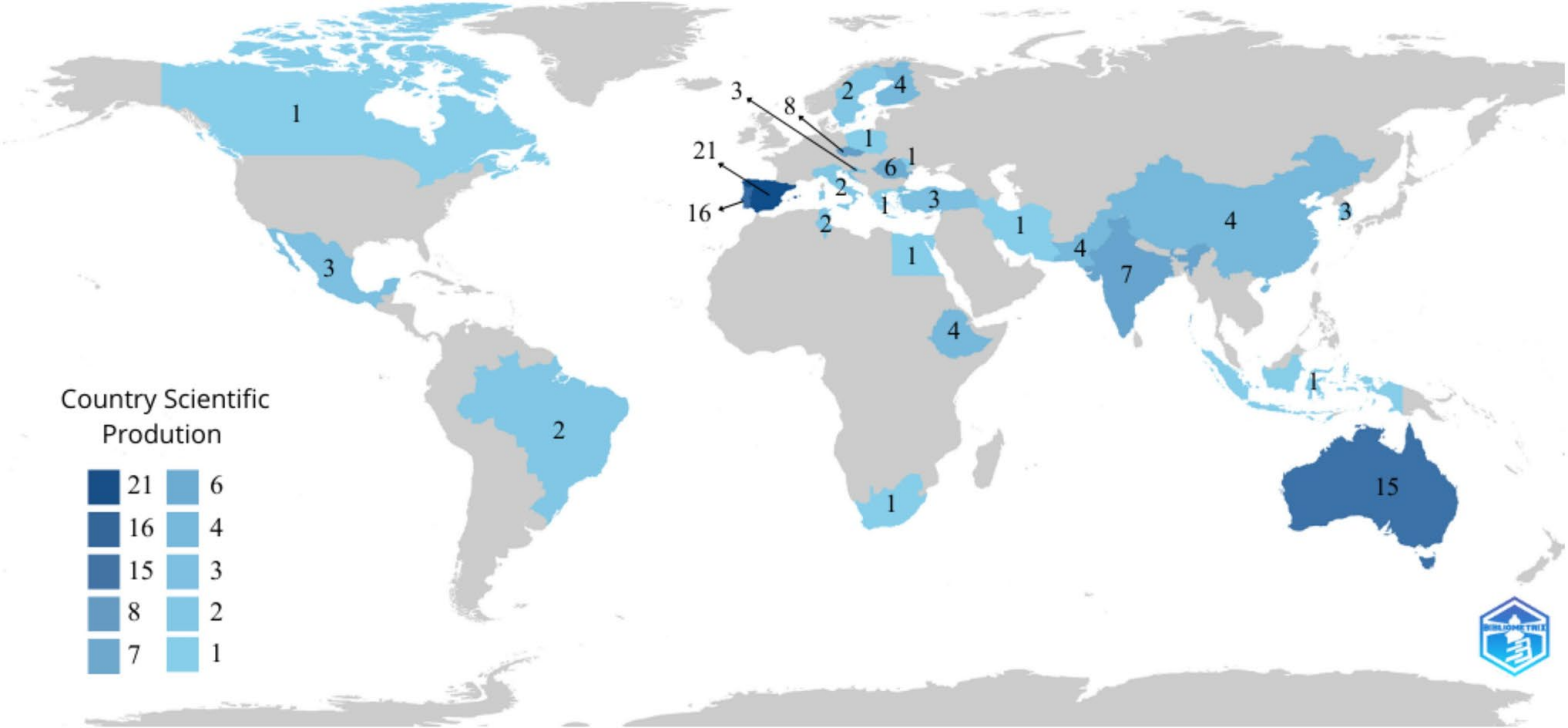
Scientific production by country based on the number of authors, considering the initial selection of articles after duplicate removal

To understand the geographical distribution of research related to this topic, Fig. 3 illustrates the percentage distribution and geographical location of global scientific production from 2015 to February 2026. The count considers the country of affiliation or nationality of the authors of the analyzed studies, rather than only the number of articles. For this reason, the total number of records exceeds the number of identified publications.

The evolution in the number of publications over this period reveals a highly internationalized research landscape, with a strong concentration in European countries and significant participation from other regions. Spain stands out as the country with the highest number of publications, with a total of 21 outputs, followed by Portugal with 16 and Australia with 15.

The strong presence of Spain does not occur in isolation but directly reflects the national regulatory context aimed at transitioning toward circular models of material management. Law 7/2022, which transposes Directive 2008/98/EC, establishes ambitious targets for the reuse and recycling of textile waste. It also requires, from 2025 onward, mandatory separate collection and prohibits the destruction of unsold surplus products. These measures, together with incentives for extended producer responsibility, create an institutional environment that encourages research, innovation, and the development of technological solutions for the valorization of end-of-life textiles (Lanz et al. 2024).

Next, an intermediate group can be observed, consisting of the Czech Republic with eight articles, India with seven, and Romania with six. The participation of these countries suggests a geographical diversification of interest in the topic, with expansion into Eastern Europe and South Asia.

China, Ethiopia, Finland, and Pakistan appear with four publications each, indicating more moderate involvement. In the case of China, although the number is relatively low considering its high overall scientific output, the result indicates that the topic has gained some attention, albeit less prominently than in the country’s more established research areas. Ethiopia and Pakistan, in turn, represent developing regions, suggesting that the topic also attracts interest in contexts where waste management and low-cost solutions are priorities.

A third group, with lower frequency, between two and three publications, includes countries such as Croatia, Mexico, South Korea, Turkey, Brazil, Italy, Sweden, and Tunisia. These results show that the topic is present across different continents, although with more occasional scientific output. In the case of Brazil, the frequency of two publications indicates an emerging field, with potential for growth given the abundance of textile waste in the country and the need for sustainable alternatives for the construction sector.

Finally, countries such as Canada, Egypt, Greece, Indonesia, Iran, Moldova, Poland, and South Africa appear with only one publication each, representing isolated efforts or research lines at an early stage of development. Although limited in volume, these records reinforce the global scope of the topic and point to opportunities for expanding research in regions that are currently underrepresented.

## Textile waste used in cementitious composites

Research on textile waste applied in cementitious composites has shown continuous growth over the past 10 years, indicating the consolidation of this topic and increasing scientific interest in the field. Among the 23 articles selected after the screening process and included in this review, Table 2 summarizes the main data extracted from the studies, providing an integrated overview of how these investigations relate to one another.

**Table 2:** Description of studies on the incorporation of textile waste in cementitious composites

Author	Composite	Waste	Format	Pre treatment	Origin	Incorporation	Curing period (days)	CS (MPa)	FS (MPa)
Bayraktar et al. (2024)	Foamed concrete	Polyester and Ceramic Powder	Fibers, diameter 20–35 µm	–	Industrial	Addition	28	8.78 (10% CP + 0.6% textile waste)	3.03 (10% CP + 0.6% textile waste)
Bartulović et al. (2022)	Concrete	Cotton	Pieces, 2 cm × 6–8 cm	Water saturated (SSD)	Industrial	Aggregate replacement	28	59.5 TX-1.7 (1.7% textile waste)	8.8 TX-S-1.7 (1.7% textile waste + silica fume)
Patrisia et al. (2025)	Structural concrete	Pond ash, polyester glass sand, and polycotton	Fibers, diameter 0.3–0.5 mm	–	Textile recycler	Aggregate replacement	28	42.6 P0.5 (20% ash, 10% glass sand, 0.5% textile waste)	6.1 P0.5 (20% ash, 10% glass sand, 0.5% textile waste)
Gamage et al. (2024)	Concrete	Nylon, polypropylene, polyester, polytrimethylene terephthalate	Fibers, 12 mm	–	Industrial	Addition	28	46.25 (Nylon T1–0.3% textile waste)	6.26 (PTT T1–0.5% textile waste)
Jayalath et al. (2024)	Concrete	95% polyester, 5% elastane	Pieces, 20 mm × 20 mm	–	Industrial	Aggregate replacement	28	43.3 (1.5% textile waste)	–
Saca et al. (2023)	Concrete	Cotton and bricks	Pieces, 20 mm × 20 mm	–	Used clothes	Textile addition/brick aggregate replacement	28	26.9 C2 (2% textile waste)	6.13 C2 (2% textile waste)
Sandanayake et al. (2025)	Concrete	Polyester, kraft fibers + silica fume (SFKF)	Fibers	–	Used clothes	Textile addition/aggregate replacement (SFKF)	28	44 KFT (2.5% textile waste + 5% SFKF)	–
Haigh (2026)	Concrete	Predominantly polyester	Fibers, 1–2 mm	–	–	Cement replacement	28	30 (44 MPa control)	–
Juradin et al. (2024)	Cement screed	Cotton	Pieces, 5–10 mm	Water saturation vs polymer binders and additives	Industrial	Addition	28	37.90 CKFW 2.50 (2.5% textile waste saturated in water)	7.10 CKFW 2.50
Tang et al. (2023)	Cement mortar	Cotton	Fibers 20, 30 and 40 mm; diameter 5–20 µm	–	Textile recycler	Addition	28	58.3 CO.8 (8% textile waste/30 mm)	11.35 CO.8
Sadrolodabaee et al. (2021c)	Fiber reinforced mortar (FRM)	69.3% cotton, 30.7% polyester	Fibers, diameter 3.6–32.1 µm	–	Textile recycler	Addition	28	112.1 TW6 (6% textile waste)	15.6 TW8 (8% textile waste)
Ayed et al. (2023)	FRM	–	Fibers (spinning processes)	–	Industrial	Aggregate replacement	28	–	3.7 M40 (40% textile waste)
Tran et al. (2023a)	FRM	Kevlar, Nomex and Cordura nylon	Fibers, 12 mm	–	Industrial	Addition	28	46.6 B2 (0.15% Kevlar/0.15% nylon)	6.62 B2
Tran et al. (2023b)	FRM	Kevlar, Nomex and Cordura nylon	Fibers (1D) 6, 12, 24 mm; pieces (2D) 12 × 3 and 6 mm	–	Industrial	Addition	28	47.5 Nyl-0.3% (1D 12 mm)	6.68 keV-0.5% (1D 12 mm)
Grings et al. (2023)	Cementitious composite	Polyester	Pieces 25 × 350 mm and 590 mm	Styrene butadiene rubber (SBR) + silica fume (SF)	Industrial	Addition	91	–	1.48 (SBR + SF)
Malchiodi et al. (2022)	Fiber reinforced cementitious composites	61% cotton, 29% cotton blend, 10% synthetics	Microfibers 3.65 ± 2.57 mm; diameter 17.28 ± 1.68 µm	Water saturation and mercerization (NaOH)	Industrial	Addition	28	–	4% textile waste (320% increase in maximum flexural load)
Sadrolodabaee et al. (2021a)	Fiber/textile reinforced mortar (FRM/TRM)	65% polyester and cotton blend; 35% linen	Fibers, various sizes (nonwoven)	–	Textile recycler	Addition	28	–	15.6 FRM (8% textile waste)/15.5 TRM (6 layers)
Sadrolodabaee et al. (2021b)	Textile reinforced cementitious	65% polyester and cotton blend; 35% linen	Fibers (nonwoven)	–	Textile recycler	Addition	28	–	15.5 TW6L (6 layers textile waste)
Sadrolodabaee et al. (2022)	Textile waste fiber reinforced cement	80% vegetable fibers (45% cotton, 35% linen), 20% polyester	Fibers (nonwoven)	–	Textile recycler	Addition	28	–	15.5 TW6L (6 layers textile waste)/0.90 (post-fire)
Sadrolodabaee et al. (2025)	Composite paving flags	70% cotton, 30% polyester	Fibers < 5 mm (nonwoven)	–	Textile recycler	Addition	28	–	8.9 (Flag-C)
Xie et al. (2024)	Electrically conductive cement composite	70% cellulose, 30% polyester	Fibers 0.5–3.5 mm; diameter 12–22 µm (nonwoven)	–	–	Addition	16	–	13
Magalhães et al. (2024)	Cement based lightweight blocks	70% wool, 25% viscose, 5% elastane	Pieces, max 30 mm	–	Industrial	Addition	28	Material deformed under continuous load absorption	–
Briga Sá et al. (2022)	Cement based lightweight blocks	70% wool, 25% viscose, 5% elastane	Pieces, max 30 mm	–	Industrial	Aggregate replacement	–	–	–

En dashes “–” indicate that the values were not reported in the studies

Textile waste comprises different categories, including clothing, household textiles, uniforms, upholstery, and industrial surplus, such as obsolete stock and off-specification products (Biyada and Urbonavičius 2025). The analysis of the reviewed studies reveals a predominance of pre-consumer waste, mainly originating from spinning, weaving, and garment manufacturing processes. This trend can be attributed to the greater homogeneity of these materials and the better traceability of their composition (Gorreta et al. 2025; Biyada and Urbonavičius 2025).

Waste from textile recycling facilities, originating from both pre- and post-consumer streams, was also identified. However, the direct use of textile products discarded by consumers was considerably less frequent. This finding highlights a contrast between the analyzed literature and the main environmental challenge faced by the sector, since post-consumer waste represents a substantial share of textile waste generated worldwide. The limited use of these materials in cementitious composites is associated with their high heterogeneity, resulting from the diversity of fibers, finishes, dyes, and contaminants, which hinders processing, standardization, and the achievement of consistent properties in the resulting materials (Gorreta et al. 2025; Biyada and Urbonavičius 2025; Karmakar et al. 2025).

Among the most frequently investigated wastes, cotton and polyester fibers stand out, reflecting the predominance of these materials in global textile production (Textile Exchange 2025). In addition to these fibers, studies involving nylon, polypropylene, wool, viscose, elastane, and high-performance technical fibers such as Kevlar and Nomex were also identified. In general, textile fibers can be classified as natural, such as cotton and wool; regenerated, produced from industrially processed natural polymers, such as viscose; and synthetic, derived from petrochemical resources, such as polyester and nylon. This compositional diversity is one of the main challenges for textile recycling, as it complicates the separation and reuse of materials (Shirvanimoghaddam et al. 2020).

Three main formats of textile waste used in cementitious composites were observed. The first group consists of short fibers randomly dispersed within the matrix, obtained by cutting or shredding the waste. The second most recurrent format corresponds to fabric cuttings or strips. In addition, some studies use nonwoven or laminated materials, applying residual fibers in multiple layers to reinforce mortars or concretes. Table 3 summarizes this comparison.

**Table 3:** Comparison of the main textile waste formats used in cementitious composites

Group	Predominant mechanism	Mechanical performance	Functional properties	Limitations
Fibers	Fiber bridging; pore refinement	Increased toughness and ductility; improved FS and tensile strength; CS tends to decrease	Improved thermal and acoustic insulation; reduced drying shrinkage	Reduced workability; fiber agglomeration at high contents (*balling*)
Cuttings	Fiber bridging; pull-out	Increased toughness and ductility; improved FS; CS tends to decrease, although slight gains may occur at low and optimized dosages	Improved thermal insulation; satisfactory fire performance; low-density materials	Reduced workability; compaction becomes more difficult
Laminates/nonwoven	Fiber bridging; pull-out; strain-hardening	Strain-hardening behavior; increased toughness and fracture energy	Improved thermal and acoustic insulation; fire resistance; reduced drying shrinkage	May require more water or superplasticizer to allow penetration into the fiber network

## Mechanical, physical, and structural properties of the composites

The analyzed studies show clear and consistent trends in the behavior of cementitious composites reinforced with textile waste, regardless of the type of matrix used. Although each study presents specific features related to the waste format, fiber type, incorporation ratio, and incorporation strategy, the underlying mechanisms observed are essentially the same, allowing an integrated interpretation of the literature.

**Fig. 4:**
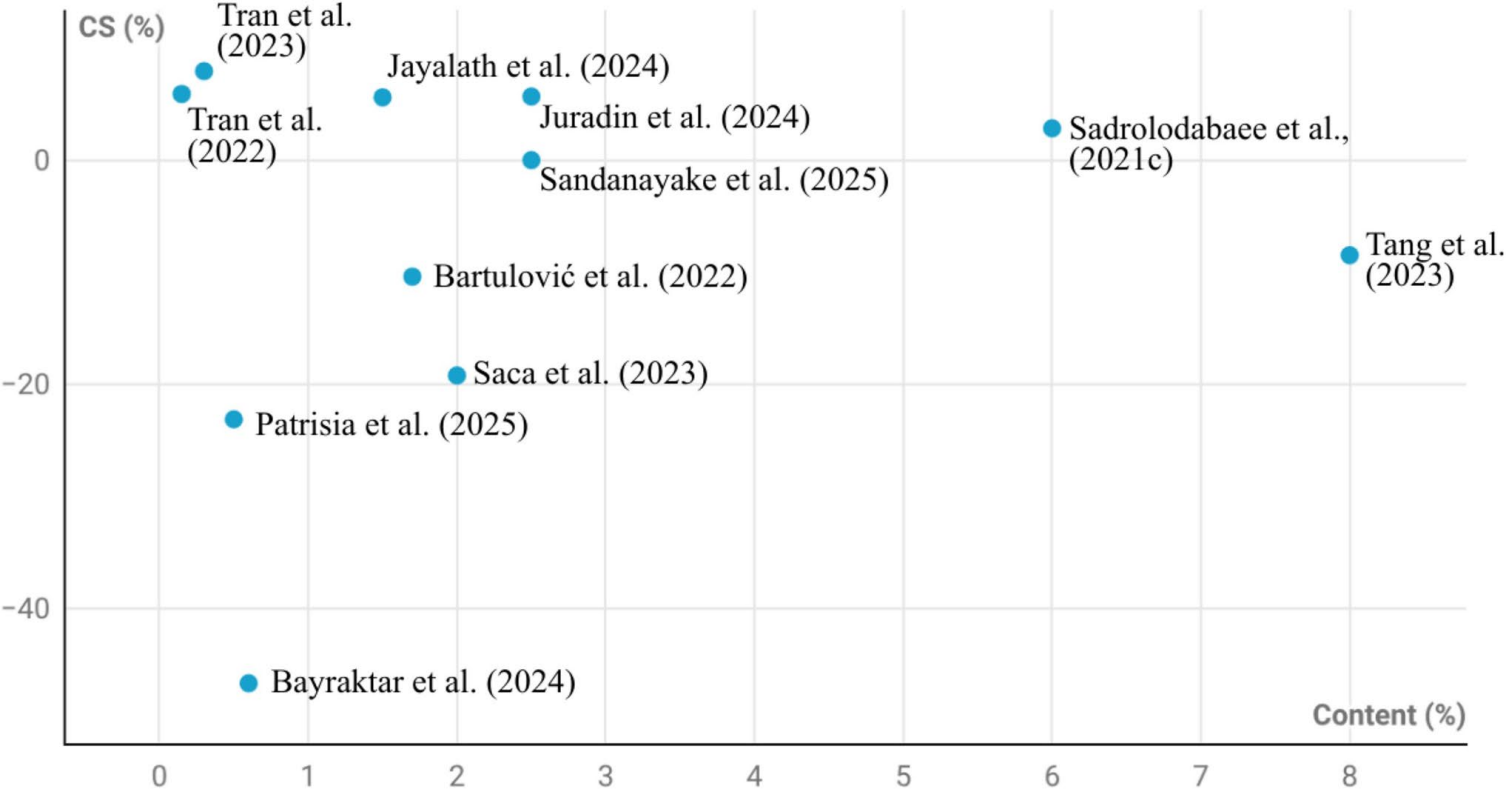
Variation in CS as a function of textile waste content

Among the evaluated properties, CS was the most frequently reported and showed highly variable behavior. In general, a predominant trend of decreasing CS was observed with increasing textile waste content incorporated into the composites. Figure 4 summarizes the variation in CS as a function of the incorporated textile waste content.

Although most studies report a reduction in CS with increasing textile waste content, the absence of a clear linear relationship between these variables suggests that the incorporated content alone is not a reliable predictor of mechanical performance.

The reduction in CS observed in mortars and concretes is related to increased porosity and void incorporation within the matrix, as well as the loss of uniformity caused by fiber agglomeration and random fiber orientation. These factors hinder compaction and weaken the fiber–matrix interface (Sadrolodabaee et al. 2021c; Patrisia et al. 2025; Haigh 2026). In mortars, the high water absorption capacity of fibers may locally alter the water-to-cement ratio and compromise the development of the cementitious matrix (Tang et al. 2023). In addition, the reduction in CS may also be associated with the low stiffness and high compressibility of textile fibers (Ayed et al. 2023). In concretes, hydrophobic fibers have been observed to result in weaker interfacial transition zones, negatively affecting the overall strength of the composite (Gamage et al. 2024).

However, some studies have shown that CS losses can be mitigated through optimization of the matrix or reinforcement characteristics. Jayalath et al. (2024) observed a progressive increase in CS with the incorporation of textile waste, attributing this result to the initial water absorption by the fibers, followed by its gradual release, acting as an internal curing agent and promoting later-age hydration. Complementarily, Patrisia et al. (2025) and Sandanayake et al. (2025) demonstrated that matrix densification through the use of pozzolans can maintain strength at levels comparable to those of the reference concrete.

In contrast to CS, FS and tensile properties showed consistent improvements in most studies that evaluated these properties. This behavior is mainly attributed to the fiber-bridging mechanism, in which the reinforcement transfers stresses across cracks, delaying crack propagation and promoting greater post-cracking deformation capacity. The interfacial slip process, or pull-out, contributes to energy dissipation and reduces the tendency toward brittle failure. As a result, the composites tend to exhibit greater toughness, energy absorption capacity, and improved post-cracking performance (Patrisia et al. 2025; Gamage et al. 2024; Tran et al. 2023a; Sadrolodabaee et al. 2021c, b).

Laminated composites showed significant increases in FS and an energy absorption capacity four times higher than that of composites with short fibers (Sadrolodabaee et al. 2021a). In laminated systems, studies indicate optimized performance in configurations with five to six reinforcement layers, while post-cracking stiffness increases proportionally with the reinforcement ratio, reinforcing the structural role of the textile layers. Among the analyzed formats, these systems proved to be the most efficient configuration for maximizing ductility, residual strength, and toughness, consolidating their potential as a promising strategy for higher-performance applications (Sadrolodabaee et al. 2021a, b, 2025). This improvement is attributed to the parallel orientation of the textile layers, which facilitates stress transfer and increases the ability to redistribute cracks throughout the material (Sadrolodabaee et al. 2021b). In this context, Xie et al. (2024) adopted a similar arrangement, replacing one of the layers with a conductive stainless-steel and polyester composite, thereby incorporating Joule-effect self-heating functionality without impairing mechanical performance.

**Fig. 5:**
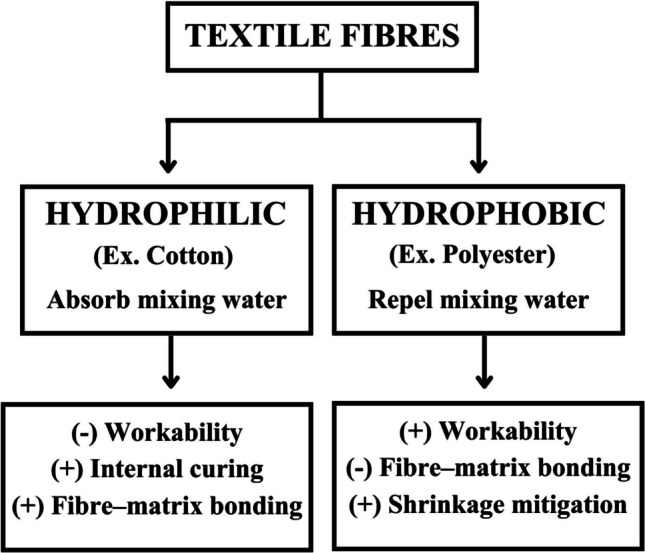
Behavior of hydrophilic versus hydrophobic fibers

Another group of successful strategies involves fiber hybridization and optimization of fiber parameters. Tran et al. (2023a) observed, in cementitious mortars, that the Kevlar/Nylon combination at a 1:1 ratio resulted in the best overall performance, with increases of up to 5.9% in CS and 13.4% in FS. It is worth noting that Kevlar and Nomex, which are hydrophilic fibers, tend to absorb water and promote additional hydration over time, whereas Nylon Cordura and other hydrophobic fibers repel water and preserve workability. The coexistence of these contrasting behaviors results in a synergistic effect that modifies the matrix microstructure and contributes to shrinkage mitigation. Figure 5 presents a conceptual model of the main mechanisms associated with the behavior of hydrophilic and hydrophobic fibers in cementitious composites.

The experimental results support this behavior. In this context, hydrophobic fibers, such as Nylon Cordura, reduced shrinkage by up to 16.2%, showing better performance than hydrophilic fibers. This difference in behavior is attributed to the fact that hydrophilic fibers, by absorbing water from the cement paste, increase the viscosity of the mixture and may promote greater pore refinement in cementitious mortars, resulting in greater volumetric deformation (Tran et al. 2023b).

This behavior is further supported by other studies. Malchiodi et al. (2022) reported substantial reductions in linear shrinkage in fiber-reinforced cementitious composites, reaching 80% with 4% residual microfibers. Gamage et al. (2024) identified systematic reductions for all types of carpet fibers evaluated in concretes, with Nylon T1 standing out, with a reduction of 22.3% at 90 days. Patrisia et al. (2025) also observed significant reductions in structural concretes, reaching 15.4% at 28 days and 23.8% at 90 days. Similarly, Bayraktar et al. (2024) reported reductions between 26 and 31% in foamed concrete with the addition of residual polyester. Sadrolodabaee et al. (2021c) observed marked reductions in shrinkage in mortars with both randomly distributed short fibers, with a 44% reduction for TW8, and laminated systems, with a 30% reduction for TW6L, indicating that different fiber morphologies exert consistently positive effects in these matrices.

In addition to mechanical properties, textile waste also significantly influences the physical properties and fresh-state behavior of cementitious composites. Overall, a reduction in workability was observed, especially in systems containing fibers with high water absorption capacity (Tang et al. 2023; Saca et al. 2023; Gamage et al. 2024; Jayalath et al. 2024; Ayed et al. 2023). This behavior is associated with water absorption by the fibers and increased internal friction within the mixture, often requiring adjustments to the water-to-cement ratio or the use of superplasticizers.

The water absorption capacity of textile waste also proved to be a determining parameter for the physical properties of the composites. In lightweight cement-based blocks, mixed wool and viscose waste showed absorption values of up to 892%, directly influencing the density and microstructure of the material. Magalhães et al. (2024) and Briga-Sá et al. (2022) observed a significant mass reduction in mixtures with higher textile waste incorporation, attributed to the high water absorption capacity of the fibers during mixing.

Another point of convergence among the studies concerns the thermal performance of cementitious composites containing textile waste. In general, fiber incorporation promotes a reduction in thermal conductivity, increasing the material’s insulation potential (Ayed et al. 2023; Malchiodi et al. 2022; Sadrolodabaee et al. 2022; Briga-Sá et al. 2022). This behavior is mainly associated with increased porosity and the intrinsically low thermal conductivity of textile fibers.

Regarding fire behavior, the results indicate that lightweight composites containing textile waste can exhibit good performance under high temperatures. Magalhães et al. (2024) observed that lightweight elements incorporating textile waste did not exhibit flammable behavior in cone calorimeter tests, contrary to the initial expectation of greater susceptibility to combustion. Similarly, Sadrolodabaee et al. (2022) found that cementitious boards reinforced with textile waste remained non-flammable and maintained structural integrity at temperatures above 950 °C. In addition, the residual post-fire FS was almost six times higher than that observed in the unreinforced reference board.

### Optimization strategies

Some of the studies investigated matrix modification strategies, such as the use of supplementary cementitious materials, aiming to mitigate the negative effects of waste incorporation by compensating for CS loss and reducing fiber-induced porosity. This can be observed in the use of pond ash by Patrisia et al. (2025), which generated pozzolanic activity in structural concrete. The pond ash promoted the secondary formation of C-A-S–H gels, ensuring continuous strength gains at later ages and compensating for the initial weakness of the composite.

Complementarily, Saca et al. (2023) found that replacing 50% of the sand with crushed bricks resulted in mechanical strengths comparable to those of the control mixture. Silica fume, in turn, proved effective in reducing water absorption (Bartulović et al. 2022). In fiber mixtures, such as kraft and textile waste, it also enabled strength equivalent to that of the control mixture due to void filling, helping to maintain high CS (Sandanayake et al. 2025). Conversely, Bayraktar et al. (2024), using ceramic powder, reported that the achieved strength remained 47% lower than that of the control, even in the optimized mixture of the developed cellular concrete.

Regarding pre-treatments, the analyzed studies indicate that the superior performance of the composites is directly associated with the quality of the fiber–matrix interface. Alkaline treatments, such as mercerization with NaOH, increased toughness and linear shrinkage performance by improving chemical and mechanical bonding with the cementitious matrix, as observed by Malchiodi et al. (2022). The impregnation of polyester fibers with SBR + SF, used by Grings et al. (2023), also stood out, enabling notable gains in toughness. This impregnation strengthened the transition zone between the textile and the cementitious matrix, thereby increasing toughness and crack resistance and confirming the improvement in adhesion between the textile reinforcement and the cementitious matrix.

In cellulosic materials, simple pre-treatments, such as water saturation, proved more effective than polymer dispersions, which tend to entrap air when used in screed mortar (Juradin et al. 2024). Bartulović et al. (2022), in turn, adopted water saturation of residual cotton fibers, followed by drainage, so that the fibers were in a saturated surface-dry condition before mixture proportioning.

### Durability and long-term performance

Although mechanical performance remains the main focus of most studies on the incorporation of textile waste into cementitious composites, durability-related properties are still considerably less investigated. Among the 23 studies included in this review, only a limited number evaluated long-term performance under environmental or aggressive exposure conditions. In addition to the small number of investigations, the lack of standardized aging and durability protocols makes direct comparisons between results difficult. Nevertheless, the available evidence suggests that the durability of these composites is mainly governed by two factors: the ability of the fibers to control cracking and shrinkage, and the chemical stability of the textile waste in the alkaline environment of the cementitious matrix.

Among the parameters associated with durability, drying shrinkage was one of the most frequently investigated properties. Overall, the incorporation of textile waste contributed to reducing shrinkage and improving crack control, with reductions of up to 80%. This behavior is mainly attributed to the fiber-bridging effect, which restricts the opening and propagation of microcracks during moisture loss, promoting greater dimensional stability of the composite (Malchiodi et al. 2022).

Accelerated aging tests, especially wetting–drying and freeze–thaw cycles, show that the durability of these composites is strongly related to the ability of the fibers to maintain their reinforcing function over time. Among the analyzed studies, wetting–drying tests were the most frequently used and were commonly conducted over 25 accelerated aging cycles. The main degradation observed occurred in toughness and energy absorption capacity, with significant reductions after aging. In general, composites reinforced with short fibers showed greater reported losses, reaching up to 42%, while laminated composites showed losses between 35 and 95% for systems with six and three layers, respectively (Sadrolodabaee et al. 2021c, b).

Similarly, composites containing synthetic fractions, especially polyester, showed better retention of mechanical properties after aging cycles, suggesting greater stability under aggressive conditions. The modulus of rupture (MOR) decreased by only 3% in composites with a higher polyester fraction, compared with losses of 20% to 25% in predominantly plant-based reinforcements (Sadrolodabaee et al. 2021c). Promising results were also observed in freeze–thaw tests, in which composites containing textile waste met the standard requirements for paving applications, with limited mass loss after repeated exposure to thermal cycles (Sadrolodabaee et al. 2025).

The long-term durability of these composites is strongly influenced by the chemical compatibility between the fibers and the cementitious matrix. Cellulosic fibers, such as cotton and flax, are more susceptible to degradation in alkaline environments. During aging cycles, the migration of hydration products, especially calcium hydroxide, or portlandite, into the lumen and cell walls promotes the progressive mineralization of the fibers, resulting in embrittlement and reduced crack-bridging capacity (Bartulović et al. 2022). As a consequence, the composite failure mechanism may evolve from a predominantly ductile behavior, characterized by fiber pull-out, to a more brittle failure mode, compromising residual toughness. In contrast, synthetic fibers, such as polyester and nylon, exhibit greater chemical stability in alkaline environments and better retention of mechanical properties after accelerated aging.

Complementary durability studies indicate that strategies such as the use of pozzolanic additions, including silica fume, are promising for densifying the matrix, reducing pore alkalinity, and mitigating long-term degradation processes (Sadrolodabaee et al. 2023, 2024). In particular, replacement levels of approximately 30% showed promising results, promoting reductions of up to 66% in calcium hydroxide content through the pozzolanic reaction and favoring the additional formation of C-S–H gel. This modification reduces the alkalinity of the pore solution, limits the migration of calcium ions into the fibers, and minimizes mineralization and embrittlement processes, especially in cellulosic reinforcements (Sadrolodabaee et al. 2023).

## Environmental analysis

Several studies have demonstrated relevant environmental benefits associated with the use of textile waste in cementitious composites, although through different methodological approaches, particularly regarding the adopted functional unit, system boundaries, and underlying assumptions. These differences hinder direct comparisons between results and limit the consolidation of robust quantitative conclusions. Table 4 summarizes the main methodological approaches identified in the reviewed studies.

**Table 4:** Comparative analysis of environmental assessment studies on cementitious composites containing textile waste

Author	Type of analysis	Functional unit	System boundaries	Adopted assumptions
Jayalath et al. (2024)	LCA focused on greenhouse gas emissions (GHG)	1 kg of recycled textile fiber	Mechanical processing, from collection to packaging	Cement and aggregates were excluded to isolate the environmental impact of textile recycling
Haigh (2026)	LCA, carbon emission reduction, impact assessment, and circular economy feasibility	1 m^3^ of concrete	Cradle-to-gate, including raw material extraction, cement production, transport, screening, and mechanical processing of waste	Uses OpenLCA and NSGA-II
Sandanayake et al. (2025)	LCA focused on carbon emissions and economic impact	1 m^3^ of concrete and 1 m^2^ of area	Cradle-to-gate, including materials, transport, and equipment	Considers “liquid emissions”, subtracting avoided impacts from not sending waste to landfill
Sadrolodabaee et al. (2022)	MIVES	1 m^2^ of façade panel	Cradle-to-gate, including manufacturing and construction	Integrates economic, environmental, and social indicators into a single Sustainability Index (SI)
Sadrolodabaee et al. (2025)	MIVES	1 m^2^ of paving slabs	Cradle-to-gate, manufacturing phase	Focuses on socio-technical and environmental indicators, with embodied carbon and energy considered
Bayraktar et al. (2024)	CO_2_ and cost estimation	1 m^3^ of foamed concrete	Cradle-to-gate, material components	Emissions from residual polyester were calculated as 1/10 of the value for virgin fiber

Bayraktar et al. (2024) directly quantified the CO_2_ emissions associated with foamed concretes and showed that residual polyester has an emission factor ten times lower than that of virgin polyester, corresponding to 0.19 and 1.93 kg CO_2_/kg, respectively. Complementarily, Jayalath et al. (2024), through a Life Cycle Assessment (LCA), determined that the production of 1 kg of recycled textile fiber generates only 0.338 kg CO_2_-eq, a value substantially lower than those reported for conventional fibers such as carbon, glass, and polypropylene. Both studies indicate that the main contributors to emissions are waste transportation and shredding. They also show that the use of textile waste reduces embodied carbon, decreases energy consumption, and replaces virgin materials with higher environmental impacts, confirming this route as a low-carbon alternative.

With a similar purpose, Sandanayake et al. (2025) conducted a cradle-to-gate assessment combined with Monte Carlo simulations and demonstrated that the Tex-crete composite can achieve net reductions of up to 3.38% in total carbon emissions when avoided emissions from landfill disposal are considered. The study also highlighted that fiber transportation and the power demand of the shredding equipment are the most critical variables controlling emissions.

Haigh (2026) carried out an LCA to assess and compare the environmental impacts of concrete incorporating waste materials, namely kraft cardboard fibers, recycled high-density polyethylene, and textile fibers. The study was also limited to a cradle-to-gate assessment and did not include scenarios related to the construction stage, in-service durability, or end-of-life. The results showed that cement reduction is the main factor driving environmental improvement, particularly regarding global warming potential (GWP). Among the materials evaluated, the composite containing kraft fibers showed the greatest reduction in carbon emissions, at 19%, followed by the material containing textile waste, at 10%. In contrast, the incorporation of recycled high-density polyethylene increased both GWP and fossil fuel depletion, despite being a recycled material. By integrating LCA with multi-objective optimization through the Non-dominated Sorting Genetic Algorithm (NSGA-II) algorithm, the authors identified textile waste as one of the most balanced alternatives in terms of environmental impact reduction and economic feasibility.

From a broader perspective, Sadrolodabaee et al. (2022) applied the MIVES methodology, or Integrated Value Model for Sustainability Assessment, which combines environmental, social, and economic criteria. In environmental terms, the material performed better than the conventional panel, mainly due to lower CO_2_ emissions, greater recyclability, and moderate resource consumption, resulting in an environmental satisfaction value of 0.71. The analysis incorporated indicators such as carbon footprint, embodied energy, material consumption, recycling potential, and environmental product declarations. Compared with the control panel, the cementitious panel reinforced with recycled textile waste achieved a Sustainability Index (SI) 24% higher, mainly driven by reduced cement use and the incorporation of recycled textile waste. The study also highlighted social benefits related to thermal and acoustic comfort, user safety, and positive impacts on recycling chains.

In a recent study by the same author, the MIVES model was applied to sustainable cementitious paving slabs produced with limestone calcined clay cement, textile waste, and recycled fine aggregates. The results indicated high socio-technical performance, 0.70/1.00, and environmental performance, 0.73/1.00, driven by low embodied carbon, 0.283 kg CO_2_-eq/kg, and embodied energy, 1.846 MJ/kg. These findings contribute to the understanding that the use of industrial by-products further improves the alignment of these materials with decarbonization goals.

In addition to global assessments, some studies focused on specific environmental mitigation strategies. Malchiodi et al. (2022), for example, evaluated the retention of fibrous microplastics and showed that incorporating up to 4 kg of textile microfibers per ton of cement paste can significantly reduce the dispersion of these contaminants into the environment. Ayed et al. (2023) demonstrated that the use of textile waste in thermal insulation solutions can reduce the operational energy consumption of buildings, strengthening the role of these materials in lowering carbon emissions associated with heating and cooling.

Finally, indirect environmental benefits were also identified, particularly through the improved thermal performance of the composites, which contributes to greater building energy efficiency and lower emissions over the service life (Bayraktar et al. 2024; Sadrolodabaee et al. 2022; Malchiodi et al. 2022; Ayed et al. 2023; Patrisia et al. 2025; Saca et al. 2023; Briga-Sá et al. 2022). In this context, converting textile waste into cementitious reinforcement not only extends the service life of fibers but also supports circular production models by reducing emissions and decreasing the volume of waste sent to landfills (Briga-Sá et al. 2022).

## Gaps and future perspectives

The analysis of the literature highlights relevant advances in the incorporation of textile waste into cementitious composites. One of the main issues identified in the literature is the scarcity of long-term durability assessments. Although mechanical, physical, and thermal properties have been widely investigated at early ages, studies evaluating the behavior of these materials under different environmental conditions and throughout their full service life remain limited (Bayraktar et al. 2024; Ayed et al. 2023; Sadrolodabaee et al. 2021b; Bartulović et al. 2022).

The applicability of textile waste in cementitious composites for non-structural elements is relatively well established. However, its extension to low- to medium-responsibility structural applications still requires further investigation, especially regarding the development of methods that allow higher fiber volumes to be incorporated without compromising workability and mechanical properties (Sadrolodabaee et al. 2021b; Gamage et al. 2024). In addition, one of the main challenges is the lack of consensus regarding the optimum textile waste incorporation content. The reviewed studies report highly variable optimum contents, ranging from low dosages below 1% to incorporation levels above 4%. This variability is directly associated with the high heterogeneity of the textile waste investigated, which includes natural fibers, synthetic fibers, polymer blends, and pre- and post-consumer waste, making it difficult to generalize the results.

At the same time, the transition of these materials to real-scale applications requires greater attention to production-related technological aspects, including applicable thicknesses, optimized geometries, cutting and molding processes, as well as architectural and construction criteria. These factors will be essential for determining the technical feasibility and effective adoption of these composites in real construction systems (Bartulović et al. 2022).

The importance of exploring different types of waste should also be emphasized, particularly by evaluating how the integration of fibers with different performances can optimize the overall behavior of the composite. This approach may also be combined with the use of pozzolanic materials as a strategy to improve the cementitious matrix (Bayraktar et al. 2024; Gamage et al. 2024; Sadrolodabaee et al. 2021b).

The advancement of new technologies also emerges as a promising pathway to accelerate the development of cementitious composites containing textile waste. Predictive models, computational modeling tools, and artificial intelligence techniques can support the optimization of mix formulations and the prediction of physical and mechanical properties, reducing experimental time and enabling the identification of more efficient combinations between cementitious matrices and incorporated waste. In addition, machine learning approaches may support comparative assessments integrating mechanical performance, environmental impacts, and economic feasibility, contributing to more robust decision-making for sustainable material design (Bayraktar et al. 2024; Sandanayake et al. 2025).

Regarding environmental analyses, the predominance of assessments restricted to the cradle-to-gate scope, combined with the high variability in functional units, system boundaries, and adopted assumptions, makes direct comparisons between studies difficult and limits the consolidation of robust quantitative conclusions.

Multicriteria sustainability models, such as MIVES, present limitations related to their dependence on criteria and weights defined according to specific regional contexts, which may compromise the generalization of results without appropriate methodological adaptations (Sadrolodabaee et al. 2022).

Furthermore, the industrial-scale implementation of these composites still depends on overcoming challenges related to waste variability, processing infrastructure, regulatory barriers, and the integration of environmental performance with economic feasibility. In this context, future studies should prioritize more comprehensive assessments, including life cycle cost analyses and investigations into large-scale recycling (Jayalath et al. 2024).

## Final considerations

Based on the literature analyzed, the main conclusions of this review can be summarized as follows:

1. Types of waste and incorporation methods: A predominance of cotton- and polyester-based textile waste was observed, mostly originating from pre-consumer sources. The main incorporation methods identified were dispersed short fibers, textile cuttings, and laminated systems, with the latter showing the best overall performance.
2. Mechanical performance: The incorporation of textile waste tends to reduce CS as the incorporated content increases, mainly due to increased porosity, compaction difficulties, and weakness of the fiber–matrix interface. In contrast, consistent improvements were observed in FS, tensile strength, and toughness, attributed to the crack-bridging mechanism and energy dissipation through pull-out. Laminated systems composed of nonwoven layers showed significantly superior mechanical performance and higher energy absorption capacity than randomly dispersed short fibers.
3. Optimization strategies and functional properties: Strategies such as fiber hybridization, the use of supplementary cementitious materials, and surface pre-treatments proved effective in mitigating mechanical losses and optimizing overall performance. The hydrophilic or hydrophobic nature of the fibers also had a relevant influence on composite behavior, affecting workability, shrinkage, and fiber–matrix interaction. In addition, the incorporation of textile waste contributed to reducing thermal conductivity, increasing the thermal insulation potential of the composites. Lightweight composites containing textile waste also did not exhibit flammable behavior and were able to maintain structural integrity at high temperatures.
4. Environmental sustainability: The studies indicate potential for reducing CO_2_ emissions, lowering embodied carbon, and contributing to the circular economy, especially when scenarios involving avoided emissions from landfill disposal are considered. Different methodological approaches have been used in these assessments, such as Life Cycle Assessment (LCA), Monte Carlo simulations, and multicriteria sustainability models. However, the diversity of methods, system boundaries, and indicators used highlights the absence of a consolidated standard, which makes direct comparison between results difficult. In addition, waste variability and the scarcity of long-term durability assessments remain relevant limitations.

Overall, the results show that textile waste has greater application potential in non-structural elements and construction systems with low to medium structural responsibility. Progress toward broader applications will depend on further studies on long-term performance, standardization of methodologies, expansion of life cycle assessments, and integration between technical performance, economic feasibility, and environmental impact.

## Data Availability

All data generated or analyzed during this study are included in this manuscript.
